# GPR120 (FFAR4) is preferentially expressed in pancreatic delta cells and regulates somatostatin secretion from murine islets of Langerhans

**DOI:** 10.1007/s00125-014-3213-0

**Published:** 2014-03-25

**Authors:** Virginia M. Stone, Shalinee Dhayal, Katy J. Brocklehurst, Carol Lenaghan, Maria Sörhede Winzell, Mårten Hammar, Xiufeng Xu, David M. Smith, Noel G. Morgan

**Affiliations:** 1 Institute of Biomedical and Clinical Sciences, University of Exeter Medical School, RILD Building, Barrack Road, Exeter, EX2 5DW UK; 2 AstraZeneca, Alderley Park, Cheshire, UK; 3 R&D, Cardiovascular and Metabolic Diseases, AstraZeneca, Mölndal, Sweden; 4 Centre for Infectious Medicine, Department of Medicine, Karolinska Institutet, Stockholm, Sweden

**Keywords:** Endocrine pancreas, FFAR4, GPR120, Insulin secretion, *n*-3 fatty acids, *n*-6 fatty acids, NEFA, Pertussis toxin

## Abstract

**Aims/hypothesis:**

The NEFA-responsive G-protein coupled receptor 120 (GPR120) has been implicated in the regulation of inflammation, in the control of incretin secretion and as a predisposing factor influencing the development of type 2 diabetes by regulation of islet cell apoptosis. However, there is still considerable controversy about the tissue distribution of GPR120 and, in particular, it remains unclear which islet cell types express this molecule. In the present study, we have addressed this issue by constructing a *Gpr120*-knockout/β-galactosidase (*LacZ*) knock-in (KO/KI) mouse to examine the distribution and functional role of GPR120 in the endocrine pancreas.

**Methods:**

A KO/KI mouse was generated in which exon 1 of the *Gpr120* gene (also known as *Ffar4*) was replaced in frame by *LacZ*, thereby allowing for regulated expression of β-galactosidase under the control of the endogenous GPR120 promoter. The distribution of GPR120 was inferred from expression studies detecting β-galactosidase activity and protein production. Islet hormone secretion was measured from isolated mouse islets treated with selective GPR120 agonists.

**Results:**

β-galactosidase activity was detected as a surrogate for GPR120 expression exclusively in a small population of islet endocrine cells located peripherally within the islet mantle. Immunofluorescence analysis revealed co-localisation with somatostatin suggesting that GPR120 is preferentially produced in islet delta cells. In confirmation of this, glucose-induced somatostatin secretion was inhibited by a range of selective GPR120 agonists. This response was lost in GPR120-knockout mice.

**Conclusions/interpretation:**

The results imply that GPR120 is selectively present within the delta cells of murine islets and that it regulates somatostatin secretion.

**Electronic supplementary material:**

The online version of this article (doi:10.1007/s00125-014-3213-0) contains peer-reviewed but unedited supplementary material, which is available to authorised users.

## Introduction

The G-protein coupled receptor 120 (GPR120, also classified as free fatty acid receptor 4 [FFAR4]; http://www.iuphar-db.org, accessed 31 January 2014) was identified by Fredriksson et al [1] and is encoded within four exons located on chromosome 10q23.33 in humans [2, 3]. De-orphanisation of this receptor revealed that the ligands for GPR120 include a variety of medium- and long-chain NEFAs, such as the *n*-3 species eicosapentaenoic acid (C20:5) and docosahexaenoic acid (DHA; C22:6). In addition, GPR120 binds various saturated fatty acids (C14–C18) as well as certain monounsaturated species with chain lengths of C16 and above [4–12].

The expression of GPR120 was confirmed in the small intestine [6] where it is thought to mediate the increased release of hormones such as cholecystokinin and glucagon-like-peptide 1 in response to the intestinal delivery of fatty acids [6, 13, 14]. Further studies have suggested that GPR120 is also expressed in a number of other tissues, including adipocytes, taste buds and certain immune cells. In these contexts, the functional role of GPR120 remains enigmatic but it has been proposed to be involved in the regulation of adipogenesis [8, 10, 15], gustation [4, 16, 17], adipocyte glucose metabolism [10] and various anti-inflammatory responses [10, 18].

A further tissue in which GPR120 has been identified by some investigators is the endocrine pancreas but its expression within islets remains contentious. Initial studies suggested that *Gpr120* (also known as *Ffar4*) mRNA is not detectable in whole pancreas, isolated islets of Langerhans or in certain pancreatic beta cell lines [6, 14, 19]. However, more recently, we and others have detected the expression of *Gpr120* mRNA in certain rat beta cell lines as well as in both primary human and rodent islets of Langerhans [20, 21]. In addition, GPR120 has been implicated as a regulator of apoptosis in human islets [22]. Thus, further studies are warranted to establish the functional role of the receptor in the islets of Langerhans.

The aim of the present investigation was to exploit a novel global knockout/knock-in approach in mice to establish more firmly whether or not GPR120 is present in the islets of Langerhans. Accordingly, a *Gpr120*-knockout/β-galactosidase (*LacZ*) knock-in (KO/KI) mouse was generated in which exon 1 of the *Gpr120* gene was replaced in frame by *LacZ*, thereby allowing for regulated expression of β-galactosidase under the control of the endogenous GPR120 promoter. These animals were used to study the distribution of β-galactosidase within mouse pancreas as a means to establish which endocrine cell types predominantly express GPR120. The functional consequences of GPR120 expression within islets were also studied.

## Methods

### Creation of KO/KI mice

A 0.567 kb fragment from exon 1 of *Gpr120* was replaced by a nuclear *LacZ* expression cassette (in frame) alongside a LoxP floxed PGK-neo selection marker in mice from a pure C57bl/6 genetic background in a colony maintained by AstraZeneca (M. Bjursell, unpublished observations). The PGK-neo marker was inserted alongside the *LacZ* gene allowing for the selection of transgenic embryos according to antibiotic resistance. Heterozygote animals were bred against an R26Cre strain to remove the LoxP floxed neo cassette as a means to yield the final knockout strain. Animals were genotyped by PCR with primers amplifying differentially sized products from the wild-type (WT) and null alleles. These studies were conducted in accordance with the Principles of Laboratory Care.

### Generation of cryosections

Pancreas and colon sections were harvested from WT and KO/KI animals, trimmed and orientated onto a cork disk, to which they were attached with cryomatrix (Thermo Scientific, Cramlington, UK) before being frozen in isopentane. Serial transverse sections (8 μm) were cut at −14 or −19°C for the colon and pancreas, respectively. The sections were air-dried for 30 min before being frozen and stained.

### β-galactosidase reporter gene assay

Pancreas or colon sections were warmed to room temperature and fixed for 10 min. Sections were washed twice in PBS prior to the addition of X-gal stain (β-galactosidase reporter gene staining kit GALS, Sigma-Aldrich, St Louis, MO, USA), incubated at 37°C for 3 h and washed in distilled water (dH_2_O) for 5 min. They were incubated in nuclear fast red (Al_2_SO_4_) for 5 min, washed in H_2_O and dehydrated: 1 × 5 min 95% industrial methylated spirit (IMS), 2 × 5 min 100% IMS and 2 × 5 min xylene. Slides were viewed and imaged under a light microscope.

### Immunofluorescence staining

Pancreas tissue was fixed in buffered 10% formalin, processed to wax blocks and sectioned onto slides (4 μm). Dual fluorescence staining was performed to determine the islet cell type in which β-galactosidase/GPR120 was expressed. Anti-insulin (1:500; Dako, Cambridge, UK), anti-glucagon (1:200; Abcam, Cambridge, UK) and anti-somatostatin (1:100; Millipore, Watford, UK) were detected using Alexa Fluor-488 conjugated anti-goat antibody (Abcam) and anti-β-galactosidase (1:1200; Abcam) with Alexa Fluor-568 conjugated anti-rabbit antibody. Citrate buffer (pH 6) was used for antigen retrieval in the case of β-galactosidase and somatostatin. Briefly, tissue sections were fixed, de-waxed and rehydrated prior to antigen retrieval (heating in a microwave at full power for 20 min followed by cooling to room temperature). The sections were blocked before the addition of primary antibodies and washed prior to the addition of secondary antibodies and DAPI nuclear stain (1:500; Thermo Scientific). Slides were mounted in Hardset Vectashield Mounting medium (Vector Laboratories, Peterborough, UK) and analysed on a Nikon Eclipse 90i Fluorescent Microscope.

### Islet isolation

Mice were terminally anaesthetised with CO_2_ then killed by cervical dislocation. Islets were isolated from WT or *Gpr120*-knockout mice by liberase digestion (1.4 mg/ml; Roche Applied Science, Indianapolis, IN, USA) and separated by centrifugation. The islets were cultured overnight in RPMI-1640 (10% FCS, 2 mmol/l l-glutamine, 100 U ml^−1^ penicillin, 100 mg ml^−1^ streptomycin, 11 mmol/l glucose) at 37°C, 5% CO_2_.

### Islet hormone secretion

Four individual islets were dispensed randomly into each well of a 24 well plate by hand and incubated in Krebs–Ringer–HEPES buffer containing 0.5 mmol/l 3-isobutyl-1-methylxanthine (IBMX) at 37°C, 5% CO_2_ for 30 min before addition of the appropriate test reagents or vehicle (DMSO, final concentration 0.3%) for a further 2 h at 37°C, 5% CO_2_. Plates were centrifuged at 1,000*g* for 1 min before the supernatant fraction was collected and frozen in readiness for hormone assay. DHA sodium salt (Sigma-Aldrich) was dissolved in water at 37°C then complexed to fatty acid free albumin (Sigma-Aldrich, 10% [wt/vol.]). The final concentration of the DHA stock was 2 mmol/l and the pH was adjusted to 7.4 before the solution was sterile filtered, aliquoted and stored at −20°C. Hormone secretion was measured after incubation of islets with 50 or 100 μmol/l DHA in 1% BSA. Control islets were incubated with 1% BSA.

### Insulin and somatostatin secretion assays

The insulin content of the incubation medium was analysed using the high-range HTRF insulin kit (62INSPEC, Cisbio Bioassays, Codolet, France) and fluorescence resonance energy transfer was measured on an Envision plate reader (PerkinElmer, Cambridge, UK). The somatostatin content of the islet incubation medium was measured using a somatostatin ELISA kit (Peninsula Laboratories, Bachem Group, Bubendorf, Switzerland) and absorbance was read at 450 nm with an Ultra Evolution XFluor4 plate reader. All standard curves were plotted using Origin 7.5 software (http://www.originlab.com).

### Islet gene expression analysis

CEL files from the GSE38642 series were downloaded from the NCBI Gene Expression Omnibus (GEO) data repository (http://www.ncbi.nlm.nih.gov/geo/query/acc.cgi?acc=GSE38642; last accessed 1 December, 2013). This dataset contains gene expression data of human pancreatic islets from 63 donors generated on Affymetrix GeneChip Human Gene 1.0 ST arrays (Affymetrix, High Wycombe, UK). Background adjustment and quantile normalisation and summarisation were computed in the Array Studio software (Omicsoft Corporation; http://www.omicsoft.com) to generate Robust Multichip Average expression values. Correlations of gene expression were calculated based on log_2_ expression values using the Pearson correlation.

### Statistical analysis

Data are expressed as the mean values ± SEM and statistical analysis was carried out using Student’s *t* test and a one- or two-way ANOVA with post hoc analysis, as appropriate. Each experiment was performed on a minimum of three separate occasions with five or more replicates in any given experiment. Differences were considered significant when *p* < 0.05.

## Results

### GPR120 is expressed in a subset of endocrine cells located peripherally in mouse islets

**Fig. 1:**
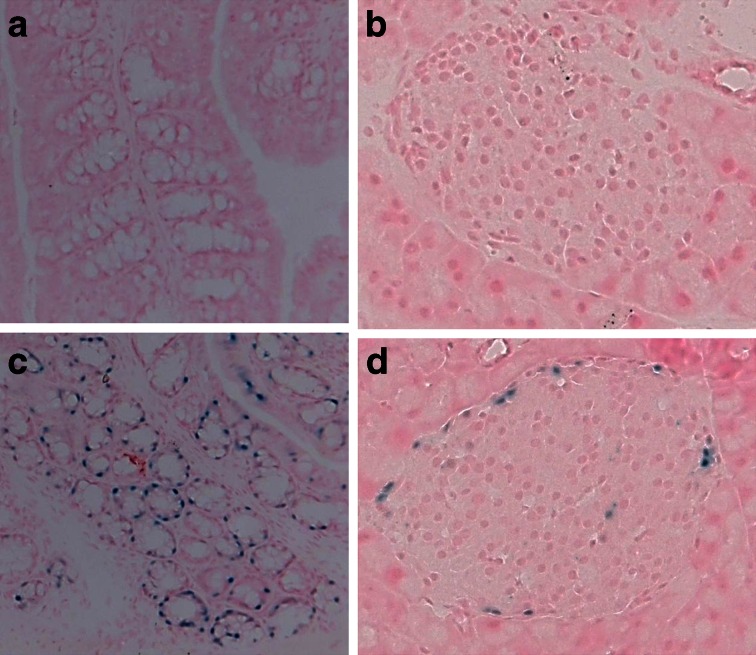
β-galactosidase (as a surrogate for GPR120) is expressed in mouse colon and peripheral cells in the islets of Langerhans. WT (**a**) colon and (**b**) pancreas, and KO/KI (**c**) colon and (**d**) pancreas cryosections were recovered and stained using an X-gal reporter kit. Images were captured under brightfield illumination and β-galactosidase positive cells are shown in blue

GPR120 is reportedly expressed in the gastrointestinal tract of mice [6, 23–25] and, in confirmation of this, sections of colon from KO/KI animals treated to reveal β-galactosidase activity, and by inference GPR120, demonstrated a distinct staining pattern within specific luminal epithelial cells (Fig. 1c). This pattern was absent from the WT animals (Fig. 1a). Importantly, similarly stained cells were also observed in the islets of Langerhans of KO/KI mice upon exposure to the substrate (Fig. 1d), but were absent in WT animals (Fig. 1b). These cells comprised only a small subpopulation of the total endocrine cell complement of each islet and were arranged peripherally within the islet mantle.

### GPR120 co-localises mainly with somatostatin in murine islets

**Fig. 2:**
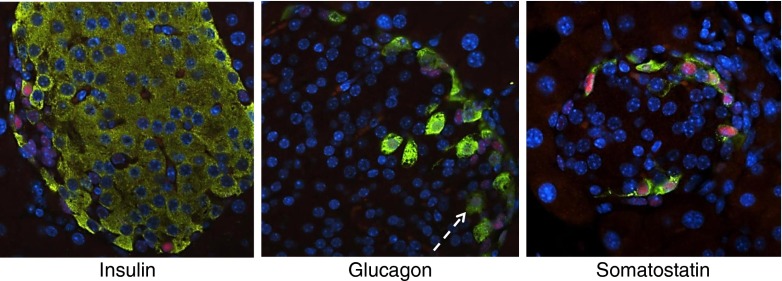
β-galactosidase (as a surrogate for GPR120) is localised mainly within the delta cells of mouse islets of Langerhans. Formalin-fixed, paraffin-embedded pancreas sections from KO/KI mice were stained by immunofluorescence for the presence of β-galactosidase (red), and each of the three islet hormones insulin, glucagon and somatostatin (green) as shown. Nuclei were stained with DAPI (blue). Images were captured on a fluorescent microscope. The white arrow indicates a cell stained positively for both β-galactosidase and glucagon

To establish the islet cell type in which β-gal (as a surrogate for GPR120) is expressed in murine islets of Langerhans, dual-immunofluorescence staining was performed in paraffin-embedded pancreas sections recovered from WT and KO/KI animals. In accord with data obtained from the enzymatic stain, immunoreactivity to β-galactosidase was detected in a subset of endocrine cells at the islet periphery in the KO/KI pancreas (Fig. 2). This was restricted to the cell nuclei, as expected, since the β-gal construct contained a nuclear localisation signal. Immunoreactivity to β-gal was not seen in the nuclei of insulin positive cells but rather appeared to co-localise predominantly with cells expressing somatostatin (Fig. 2). In addition, a minority of glucagon positive cells had β-gal positive nuclei (Fig. 2, white arrow). Among 197 alpha cells examined, 29 were positive for β-gal (14.7%). By contrast, examination of >150 somatostatin positive cells in multiple islets revealed that all were also positive for β-gal.

### Effects of GPR120 ligands on somatostatin secretion

**Fig. 3:**
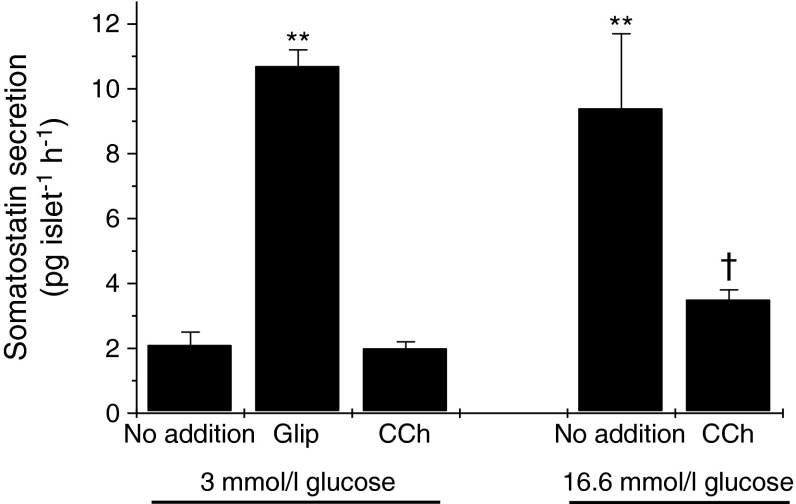
Effects of glucose, Glip and CCh on somatostatin secretion from murine islets of Langerhans. Mouse islets were isolated, cultured overnight and incubated for 2 h with 3 mmol/l or 16.6 mmol/l glucose in the presence or absence of 100 nmol/l Glip or 500 μmol/l CCh as shown. The medium was supplemented with 500 μmol/l IBMX. The supernatant fraction was sampled and somatostatin secretion was measured by ELISA. Data are presented as mean values ± SEM and the experiment was repeated on a minimum of three separate occasions. **p < 0.01 vs 3 mmol/l glucose alone; ^†^ *p* < 0.05 vs 16.6 mmol/l glucose alone

Since GPR120 appears to be expressed predominantly in delta cells, the effects of selective ligands on somatostatin secretion were investigated. Initially, the responses of isolated murine islets to known stimuli were studied as a means to validate the hormone assay system. Accordingly, it was found that a rise in the glucose concentration from 3 to 16.6 mmol/l enhanced somatostatin secretion from isolated islets (Fig. 3). Similarly, incubation with 100 nmol/l glipizide (Glip) also augmented somatostatin secretion from islets incubated with 3 mmol/l glucose and the levels achieved were similar to those found upon exposure to 16.6 mmol/l glucose. By contrast, the muscarinic cholinergic agonist carbachol (CCh) failed to alter the rate of somatostatin secretion from islets incubated with 3 mmol/l glucose but significantly attenuated somatostatin secretion from islets incubated with 16.6 mmol/l glucose (Fig. 3).

**Fig. 4:**
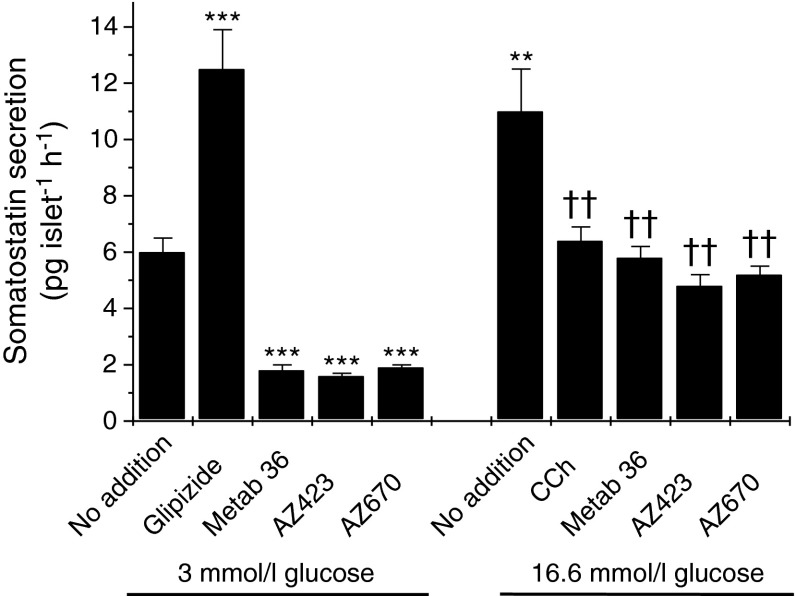
Effects of selective GPR120 agonists on somatostatin secretion from isolated mouse islets of Langerhans. Mouse islets were isolated, cultured overnight and incubated for 2 h with 3 mmol/l or 16.6 mmol/l glucose in the absence or presence of 100 nmol/l Glip, 500 μmol/l CCh or 30 μmol/l of each of three GPR120 agonists (Metabolex 36 [Metab 36], AZ-423 and AZ-670). The incubation medium was supplemented with 500 μmol/l IBMX. Following incubation, the supernatant fraction was collected and analysed for somatostatin secretion by ELISA. Data are presented as mean values ± SEM and the experiment was repeated on a minimum of three separate occasions. **p < 0.01 vs 3 mmol/l glucose alone; ***p < 0.001 vs 3 mmol/l glucose alone; ^††^ *p* < 0.01 vs 16.6 mmol/l glucose alone

In order to investigate whether or not the activation of GPR120 exerts an influence on somatostatin secretion, three structurally distinct GPR120 agonists were employed. Two of these compounds (AZ-423 and AZ-670) are proprietary to AstraZeneca while the third (Metabolex 36; CymaBay Therapeutics (formerly Metabolex), San Francisco, CA, USA) has been developed independently as a selective agonist of GPR120 [12]. All three agonists have >100-fold selectivity for GPR120 vs murine GPR40 and their selectivity is summarised in ESM Table 1. In multiple experiments, all three of the agonists tended to lower the basal rate of somatostatin secretion achieved when islets were incubated with 3 mmol/l glucose. Moreover, under stimulating conditions (i.e. in the presence of 16.6 mmol/l glucose) each consistently caused a significant reduction in hormone release (Fig. 4).

**Fig. 5:**
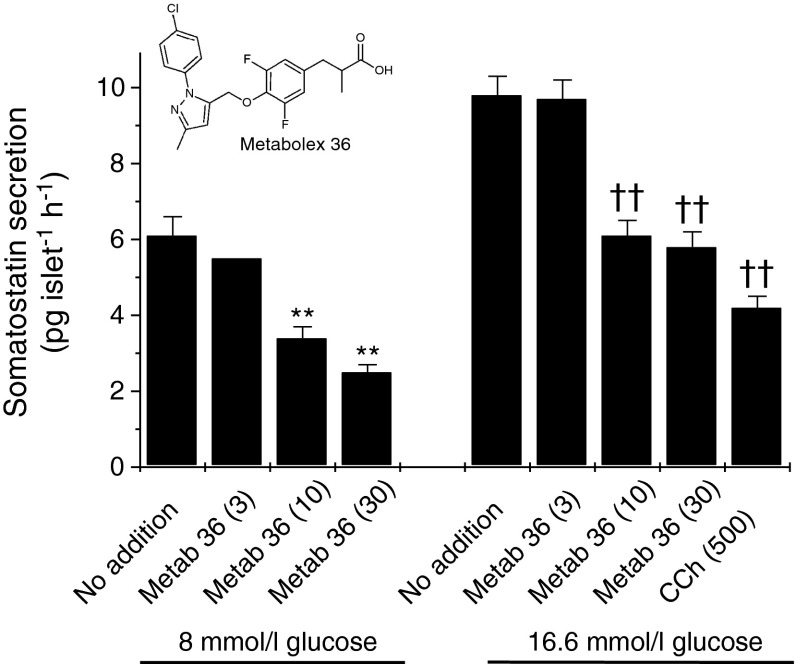
Effects of the GPR120 agonist Metabolex 36 on glucose-induced somatostatin secretion from isolated mouse islets of Langerhans. Mouse islets were isolated, cultured overnight and incubated for 2 h in the presence of 8 mmol/l or 16.6 mmol/l glucose in the absence or presence of 500 μmol/l CCh or increasing concentrations of Metabolex 36 (Metab 36) (3, 10 and 30 μmol/l). The incubation medium was supplemented with 500 μmol/l IBMX. The supernatant fraction was sampled and somatostatin secretion was measured by ELISA. Data are presented as mean values ± SEM and the experiment was repeated on a minimum of five separate occasions. **p < 0.01 vs 8 mmol/l glucose alone; ^††^ *p* < 0.01 vs 16.6 mmol/l glucose alone

In view of these results, the effects of Metabolex 36 were studied in more detail (Fig. 5). This reagent inhibited glucose-induced somatostatin secretion in a dose-dependent manner when islets were incubated under either maximal stimulating conditions (16.6 mmol/l) or with an intermediate glucose concentration (8 mmol/l). Maximal inhibition was achieved with 30 μmol/l of the ligand. By contrast, Metabolex 36 had no effect on insulin secretion under either basal or glucose stimulating conditions.

### Effect of a GPR120 agonist on glucose-induced somatostatin secretion from the islets of Gpr120-knockout mice

**Fig. 6:**
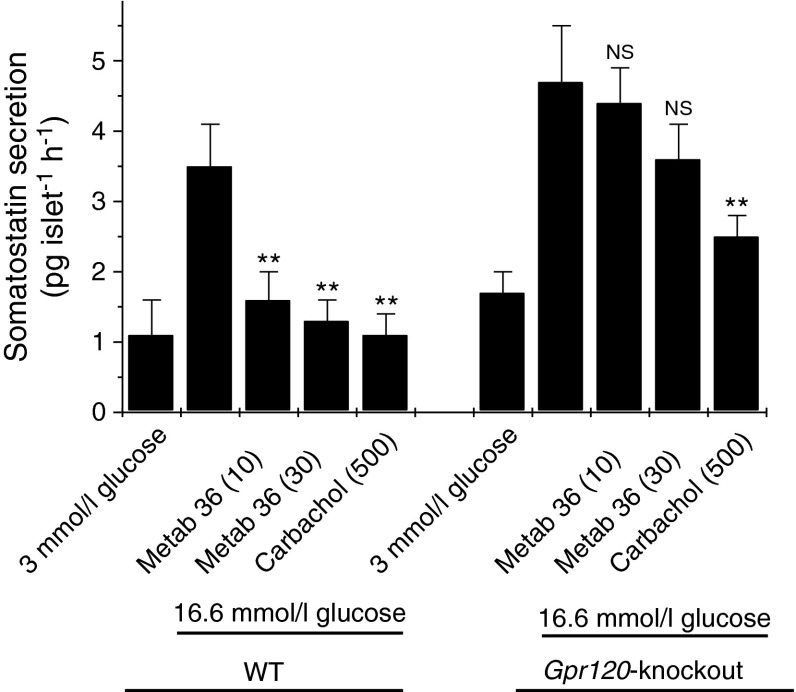
The inhibitory effects of Metabolex 36 on somatostatin secretion are lost in *Gpr120*-knockout animals. Islets were isolated from 16 WT and 16 *Gpr120*-knockout animals, cultured overnight and treated with 3 or 16.6 mmol/l glucose in the absence or presence of 500 μmol/l CCh or Metabolex 36 (Metab 36) (10 or 30 μmol/l) for 2 h. The incubation medium was supplemented with 500 μmol/l IBMX. After this time, the supernatant fraction was sampled and somatostatin secretion was analysed by ELISA. Data from individual animals were recorded separately and are presented from a representative experiment (*n* = 5 for each data point). **p < 0.01 vs 16.6 mmol/l glucose alone; NS, not significantly different from 16.6 mmol/l glucose alone

To confirm that the actions of Metabolex 36 reflect its ability to activate GPR120, the somatostatin secretion experiments were repeated in islets from KO/KI animals. Islets were isolated from 16 WT and 16 *Gpr120*-knockout mice on four separate occasions, and somatostatin secretion was monitored (Fig. 6). As seen previously, Metabolex 36 attenuated glucose-induced somatostatin secretion from the islets of WT animals. KO/KI animals responded normally to glucose, with a two- to threefold increase in somatostatin secretion when the glucose concentration was raised from 3 to 16.6 mmol/l, but in islets from these mice Metabolex 36 failed to significantly reduce glucose-induced somatostatin secretion (Fig. 6).

### Effects of pertussis toxin on the actions of Metabolex 36 in mouse islets

**Fig. 7:**
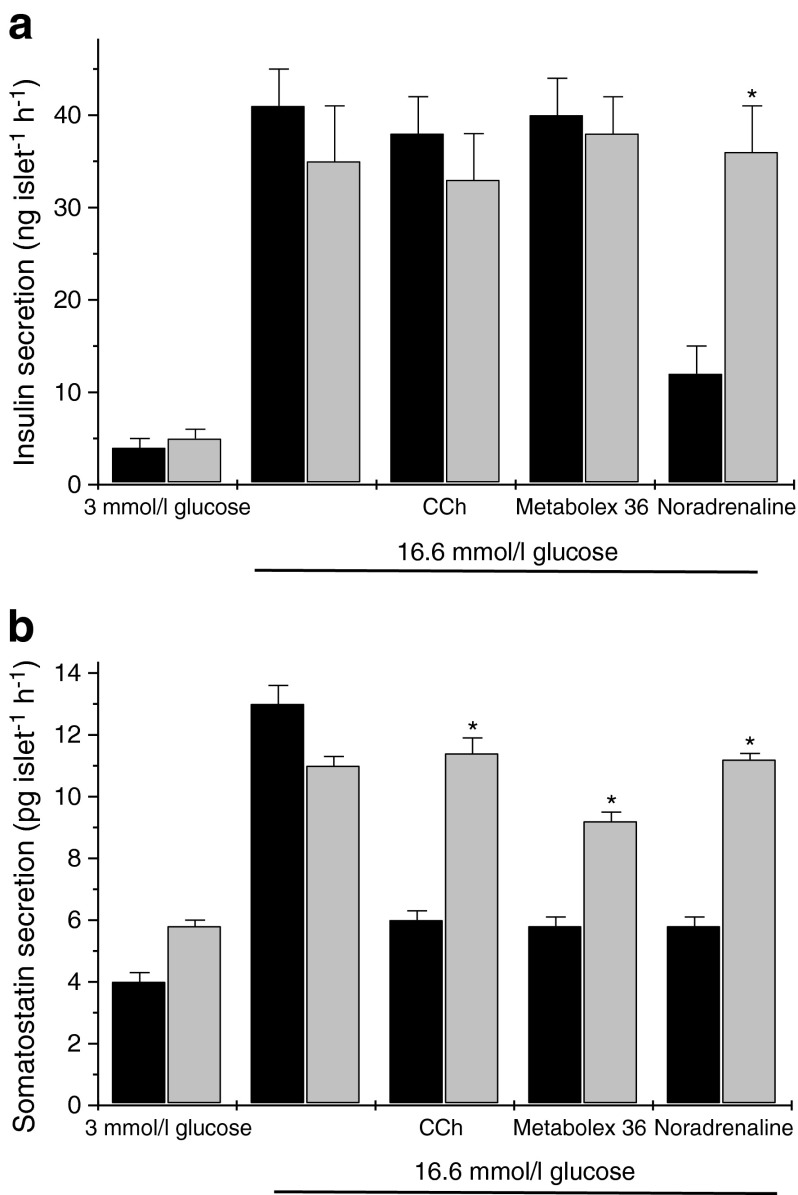
Effects of Ptx on agonist-regulated insulin or somatostatin secretion from mouse islets of Langerhans. Mouse islets were isolated and cultured overnight in the presence (light grey bars) or absence (black bars) of 100 ng/ml Ptx. They were then exposed for 2 h to media containing 3 mmol/l or 16.6 mmol/l glucose in the absence or presence of 500 μmol/l CCh, 30 μmol/l Metabolex 36 or 1 μmol/l noradrenaline (norepinephrine). The incubation medium was supplemented with 500 μmol/l IBMX. Following incubation, the supernatant fraction was sampled and assayed for either (**a**) insulin or (**b**) somatostatin secretion. Data are presented as mean values ± SEM and the experiment was repeated on a minimum of three separate occasions. *p < 0.05 compared with the relevant control in the absence of Ptx

In order to probe the signal transduction mechanism by which GPR120 exerts its effects in murine islets, pertussis toxin (Ptx) was employed. This agent ADP-ribosylates the alpha subunits of G-proteins belonging to the Gi/Go family, thereby inhibiting their signalling functions. As a positive control for the actions of Ptx, both insulin and somatostatin secretion were measured in parallel and various agents were employed that are known to influence hormone secretion in a Ptx-sensitive manner. Incubation of murine islets with 16.6 mmol/l glucose resulted in a 9–10-fold increase in insulin secretion and the magnitude of this response was not influenced by prior incubation with 100 ng/ml Ptx (Fig. 7a). Addition of the alpha2-adrenoceptor agonist, noradrenaline (norepinephrine; 1 μmol/l) significantly attenuated glucose-induced insulin secretion and, as expected from previous work [26], this response was abrogated upon preincubation with Ptx. Similarly, although Ptx had no significant effect on rates of somatostatin secretion from islets incubated with either 3 mmol/l glucose or 16.6 mmol/l glucose alone; it reduced the inhibitory actions of CCh on somatostatin secretion seen when islets were treated with 16.6 mmol/l glucose (Fig. 7b). Importantly, Ptx also attenuated the inhibitory effect of Metabolex 36 on glucose-induced somatostatin secretion.

### Effects of DHA on insulin and somatostatin secretion from mouse islets

**Fig. 8:**
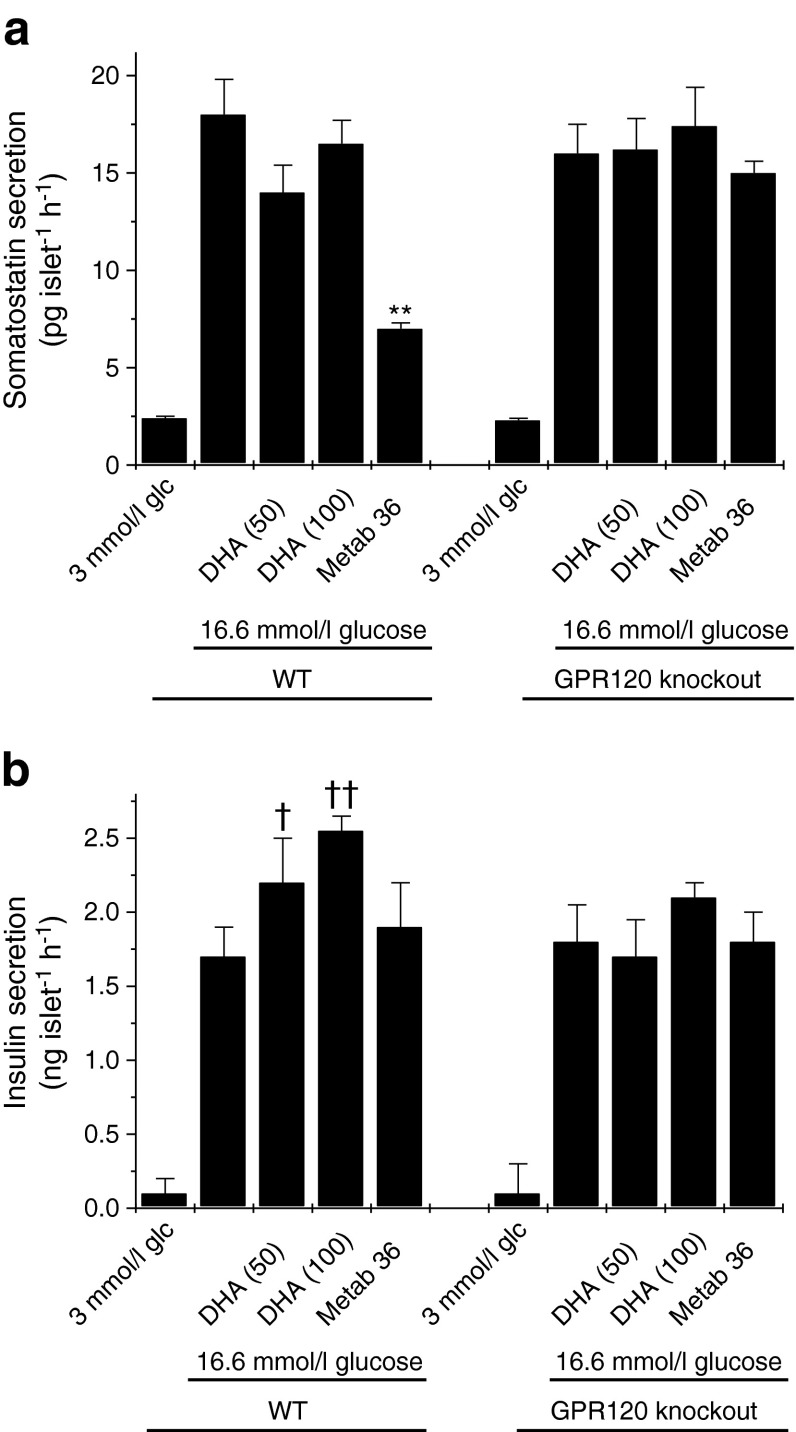
Effects of DHA on somatostatin or insulin secretion from islets of WT or *Gpr120*-knockout animals. Islets were isolated from three WT and three *Gpr120*-knockout animals, cultured overnight and treated with 3 or 16.6 mmol/l glucose (glc) in the absence or presence of DHA (50 or 100 μmol/l) or Metabolex 36 (Metab 36) (30 μmol/l) for 2 h. The incubation medium was supplemented with 500 μmol/l IBMX. After this time, the supernatant fraction was sampled and assayed for either (**a**) somatostatin or (**b**) insulin secretion. Data from each animal were recorded separately and are presented from a representative experiment (*n* = 5 for each data point). **p < 0.01 vs somatostatin secretion in the presence of 16.6 mmol/l glucose alone; ^†^ *p* < 0.05 vs insulin secretion in the presence of 16.6 mmol/l glucose alone; ^††^ *p* < 0.01 vs insulin secretion in the presence of 16.6 mmol/l glucose alone

In view of the finding that selective, synthetic GPR120 agonists cause inhibition of somatostatin secretion it was of interest to also test the effects of a putative physiological ligand. Accordingly, the response to the *n*-3 fatty acid DHA was studied (Fig. 8). In contrast to Metabolex 36, DHA failed to attenuate glucose-induced somatostatin secretion from islets isolated from either WT or *Gpr120*-knockout mice (Fig. 8a). Importantly, despite the lack of response to DHA, the inhibition mediated by Metabolex 36 was retained in WT islets in these experiments and was lost after knockout of GPR120. To verify that DHA was active in the islets under the conditions employed, insulin secretion was also measured in the same samples. DHA dose-dependently potentiated insulin secretion from WT islets but it was ineffective in islets lacking GPR120 (Fig. 8b).

### Expression patterns of GPR120 in human islets

Having established that, in murine islets, GPR120 is primarily expressed in delta cells and that it may regulate somatostatin secretion, it was considered important to extend this analysis to determine the expression patterns of GPR120 in human islet cells. In practice, this proved problematic because, although a variety of antisera that are thought to be reactive against GPR120 are available commercially, in our study the specificity of these reagents could not be verified. Thus, we adopted a different approach by analysing the correlation between GPR120 expression at the mRNA level with other genes that are expressed differentially in the various endocrine cell types present within islets.

The GSE38642 dataset contains expression levels of 28,869 transcripts in islets from each of 63 donors (54 nondiabetic and nine type 2 diabetic subjects [22]). Correlations were made between the expression of various genes present in individual endocrine cell types with that of GPR120 (Probeset 7929344) to establish which cell type is most strongly correlated with expression of GPR120 (Table 1). The most robust correlation (*r* = 0.66, *p* = 5.2 × 10^−9^) was between GPR120 and somatostatin (Probeset 8092682). Correlations with other endocrine cell markers were weaker: insulin *r* = 0.60, *p* = 2.3 × 10 ^−7^; glucagon *r* = 0.41, *p* = 0.0009; ghrelin *r* = 0.40, *p* = 0.0011; pancreatic polypeptide *r* = 0.16, *p* = 0.21 (Table 1). Similarly, marker genes for duct, acinar, endothelial, pancreatic stellate and myeloid cells were detectable but had correlation coefficients with GPR120 <−0.089 (Table 1).

**Table 1:** GPR120 expression correlates most highly with the expression of somatostatin in the human pancreas

Marker gene product	Pancreatic cell type	Correlation coefficient
Glucagon	Alpha	0.41
Insulin	Beta	0.60
Somatostatin	Delta	0.66
Pancreatic polypeptide	PP	0.16
Ghrelin	Epsilon	0.40
Elastin	Acinar	−0.44
Keratin 7	Ductal	−0.42
Kinase insert domain receptor	Endothelial	−0.09
Vimentin	Pancreatic stellate cell	−0.037

Gene expression analysis was used to correlate the expression of GPR120 with other markers of pancreatic endocrine and exocrine cells in samples from each of 63 individual pancreas donors within the GEO dataset GSE38642. The strongest correlation was with somatostatin

## Discussion

The expression pattern of GPR120 within the endocrine pancreas remains controversial with initial reports suggesting its absence from human islets of Langerhans as well as from clonal mouse pancreatic beta cells [6, 19]. By contrast, we [21] as well as Gotoh et al [15] and Kebede et al [20] have reported that GPR120 is present at the mRNA level in rodent pancreas beta cell lines and in rat or mouse islets. In addition, Taneera et al [22] have reported GPR120 expression in human islets. In the present work, we confirm the outcomes of these latter studies by demonstrating the expression of *GPR120* mRNA in mouse and human islets. However, we also show that the receptor is not present in the majority of islet endocrine cells and, consistent with the initial reports of Hirasawa et al [6] we find no evidence that it is present in mouse beta cells (although, as noted above, in earlier work we detected GPR120 in various clonal rat beta cells, suggesting either that species differences exist or that clonal rat beta cells are unrepresentative of primary cells with respect to GPR120 expression).

Despite our failure to detect GPR120 in mouse beta cells, the use of a KO/KI approach revealed that the GPR120 promoter is functionally active within a subset of endocrine cells located peripherally within the mantle of mouse islets. This was confirmed both by a colourimetric assay, in which the activity of the enzyme was detected directly in unfixed cryosections, and by immunofluorescence analysis using an antiserum directed against β-gal. Moreover, the immunopositive cells were co-stained by an antiserum directed against somatostatin suggesting that they were delta cells. Taken together, these data imply that GPR120 is preferentially localised within the delta cells of mouse islets but that it is absent from beta cells. A small proportion of glucagon positive islet cells (∼15%) also expressed β-gal, which is consistent with a recent report that the clonal alpha cell line, alpha-TC1, may express GPR120 [27].

In order to verify the situation in human islets, we correlated the abundance of *GPR120* mRNA with other known endocrine cell markers. This revealed that expression of GPR120 was more strongly correlated with somatostatin than with either insulin or glucagon in human islets. Hence, although we cannot conclude that human alpha and beta cells are devoid of GPR120, these results imply that the receptor is enriched in the delta cell population in human islets, consistent with our findings in mice.

Having established that GPR120 is expressed predominantly in the somatostatin positive delta cells of murine islets of Langerhans, further studies were initiated to examine the functional role of GPR120 in these cells. The delta cells typically comprise 5–10% of the endocrine population of mouse islets and they synthesise and secrete somatostatin-14, an isoform that accounts for approximately 5% of circulating somatostatin [28, 29]. Accordingly, we employed an assay system to monitor somatostatin-14 secretion from mouse islets and examined the effects of various stimuli, including three selective GPR120 agonists. The results revealed that a rise in glucose concentration from 3 to 16.6 mmol/l was associated with a two- to threefold increase in somatostatin secretion and that the sulfonylurea Glip also promoted somatostatin secretion. These data are consistent with a substantial body of earlier work [29–39]. In addition, we observed that the muscarinic cholinergic agonist CCh inhibited glucose-induced somatostatin secretion, in accord with Hauge-Evans et al [33, 34]. Importantly, it should be noted that muscarinic cholinergic receptor isoforms are expressed differentially in beta and delta cells and, as a consequence, their responses to parasympathetic stimulation are different. Beta cells express the M3 isoform (whose agonists stimulate insulin secretion), whereas the M2 and M4 isoforms are involved in the parasympathetic inhibition of somatostatin secretion from delta cells [33]. In our studies, each of the three GPR120 agonists tested caused a marked inhibition of glucose-induced somatostatin secretion. This implies that GPR120 may be coupled to a signalling pathway that mediates inhibition of somatostatin secretion from delta cells. The GPR120 agonists did not alter the rate of insulin secretion from mouse islets under any condition studied. This is consistent with the observation that GPR120 is not expressed in beta cells, but it also implies that the paracrine effects of somatostatin on the beta cells (which might be expected to have been relieved during incubation with GPR120 agonists, thereby leading to enhanced insulin secretion) were only minimally effective under the conditions of static incubation used in the present experiments.

To verify that the effects observed were due to agonism at GPR120, islets from *Gpr120*-knockout animals were utilised and the responses to the GPR120 agonist Metabolex 36 were compared with those in WT mice. The results revealed that although the GPR120 agonist consistently inhibited glucose-induced somatostatin secretion from WT islets, it was ineffective in islets from *Gpr120*-knockout mice.

Previous studies have suggested that the C16 saturated fatty acid palmitate inhibits somatostatin secretion [40] but it is not known whether or not this reflects agonism at GPR120 since palmitate exerts pleiotropic effects in islet cells. Thus, we tested the effects of DHA, an *n*-3 fatty acid, which has been proposed as a physiological GPR120 agonist [2, 10, 11]. However, unlike Metabolex 36, DHA (up to 100 μmol/l) failed to attenuate glucose-induced somatostatin secretion. This may be because DHA exerts multiple effects on islet cells (including both metabolic and receptor-mediated responses) but, whatever the reason, these results emphasise the value of selective, synthetic agonists for delineating the role of GPR120 in islets. As confirmation that DHA was active under the conditions employed, insulin secretion was also studied and it was shown that the fatty acid dose-dependently potentiated insulin secretion from WT murine islets (Fig. 8b). However, it is interesting to note that DHA failed to increase insulin secretion from the *Gpr120*-knockout islets. The reasons for this are unclear but the results could be taken to imply that activation of GPR120 plays a facilitatory role in mediating the enhanced insulin secretion. However, this conclusion is not supported by data obtained with the more selective GPR120 agonist Metabolex 36, which failed to promote insulin secretion under any condition studied (Figs 7a and 8b).

Using transfection models a number of different coupling mechanisms have been proposed for GPR120. Initially, it was suggested that GPR120 couples to the G_αq_ G-protein as HEK293 cells stably expressing the receptor responded to the GPR120 agonist linolenic acid, with an increase in intracellular Ca^2+^ and induction of downstream signalling molecules of the extracellular signal-regulated kinase/phosphatidylinositol 3-kinase signalling pathway [6]. Similar effects were also seen in the human intestinal cell line STC-1, where agonism at GPR120 was protective against serum withdrawal, and these protective effects were lost in the presence of a phospholipase C inhibitor U73122 [41]. In our studies, the involvement of Gq has not been tested directly but we consider it unlikely that this mechanism operates in mouse delta cells since this would result in enhanced hormone secretion by virtue of the increased cytosolic Ca^2+^ levels. Rather, we noted a decline in somatostatin secretion. Importantly, it should be noted that the calculated potency of each of the various GPR120 agonists has been derived mainly from analyses in which the activation of Gq was the functional readout. Since we now show that, in islets, the responses are unlikely to be mediated via Gq it is possible that their potencies may be altered in the islet system. This might, therefore, account for the finding that 3 μmol/l Metabolex-36 was ineffective as an inhibitor of somatostatin secretion (Fig. 4), whereas this concentration is some threefold greater than its calculated half maximal effective concentration (EC_50_) (ESM Table 1).

From our studies it appears that GPR120 may couple, at least in part, to the Ptx-sensitive G_αi_ in murine islets. Thus, we observed that preincubation of islets with Ptx significantly attenuated the inhibitory effects of Metabolex-36 on glucose-induced somatostatin secretion in a similar manner to that seen previously with CCh [33]. Since Gi is typically coupled to adenylate cyclase these results imply that GPR120 is likely to reduce cAMP levels in murine islets, although it should be noted that the inhibition of hormone secretion via Gi may also involve a range of other mechanisms [42]. An additional consideration is that in order to facilitate the measurement of somatostatin secretion the incubation medium was supplemented with IBMX, which would be expected to mediate a rise in intracellular cAMP. Hence, any attenuation of cAMP synthesis would be manifest as a reduction in stimulated somatostatin secretion, and we deduce that this is the mechanism by which GPR120 agonists exert their effects. Overall, therefore, these data strongly suggest that GPR120 is coupled to Gi in mouse delta cells and that this leads to reduced rates of somatostatin secretion under conditions of glucose stimulation.

## Electronic supplementary material

Below is the link to the electronic supplementary material.

ESM Table 1 (PDF 28 kb)

## Abbreviations

CCh: Carbachol
DHA: Docosahexaenoic acid
FFAR4: Free fatty acid receptor 4
Glip: Glipizide
GPR120: G-protein coupled receptor 120
IBMX: 3-Isobutyl-1-methylxanthine
KO/KI: *Gpr120*-knockout/β-galactosidase (*LacZ*) knock-in
Ptx: Pertussis toxin
WT: Wild-type
