# Supplementary material for: GPR120 (FFAR4) is preferentially expressed in pancreatic delta cells and regulates somatostatin secretion from murine islets of Langerhans
Source: Diabetologia. 2014 Mar 25;57(6):1182–91. doi: 10.1007/s00125-014-3213-0 (PMC4018485; doi:10.1007/s00125-014-3213-0)
Supplement: Supplementary file 1 — (PDF 28 kb) [file 125_2014_3213_MOESM1_ESM.pdf]

Supplementary table (S1). Potencies of selective GPR120 agonists at either GPR120 (FFA4) or GPR40 (FFA1). Compounds were analysed using either fluorescent imaging plate reader (FLIPR) or label-free dynamic mass redistribution (DMR) technologies and the respective EC_{50} values (µmol/l) calculated.

**Compound**	**EC_{50} at GPR120**		**EC_{50} at GPR40**
	DMR	*FLIPR*	*FLIPR*
Metabolex 36	0.99µmol/l	1.17µmol/l	>100µmol/l
AZ670	0.41µmol/l	1.09µmol/l	>30µmol/l
AZ423	0.51µmol/l	1.60µmol/l	>100µmol/l
